# Evaluation of the anaesthetic properties and tolerance of 1:100,000 articaine versus 1:100,000 lidocaine. A comparative study in surgery of the lower third molar

**DOI:** 10.4317/medoral.17414

**Published:** 2011-12-06

**Authors:** Natalia Martínez-Rodríguez, Cristina Barona-Dorado, María Martín-Arés, Jorge Cortés-Bretón-Brinkman, José M. Martínez-González

**Affiliations:** 1 Oral surgery. Department of Orofacial Surgery and Implantation of the University Hospital of Madrid; 2 Associate professor of Oral Surgery at U.C.M. and assistant director of the Master’s Program in Oral Surgery and Implantation of the University Hospital of Madrid; 3 Associate Professor of Prosthetics. School of Health Sciences. King Juan Carlos University; 4 Professor of Oral Maxillofaciale at U.C.M. and Chief of the Department of Oral Surgery and Implantation of the University Hospital of Madrid

## Abstract

Objectives: To evaluate the anaesthetic properties and tolerance of articaine versus lidocaine at equal vasoconstrictor concentration. Study Design: A total of 96 male and female patients who underwent surgical treatment of the lower third molar participated. Patients were randomly assigned to articaine hydrochloride with epinephrine 1:100,000 and lidocaine hydrochloride with epinephrine 1:100,000. The variables analysed were latency period, duration of anaesthetic effect, tolerance and adverse reactions. Results: Both the latency period and the duration of anaesthetic effect were greater for articaine, although the differences were not statistically significant. Latency: mean difference of 2.70 ± 2.12 minutes (95%CI of -1.51 minutes - 6.92 minutes). Duration: mean difference of -33 minutes 5 seconds ± 31 minutes (95% CI -1 hour 35 minutes - 29 minutes). There were 4 adverse events that did not require the patients to be withdrawn from the study. Conclusions: The anaesthetics in this study have very similar properties for use in surgery and have demonstrated a good safety and tolerability profile

** Key words:** Articaine, lidocaine, vasoconstrictor, adverse reactions.

## Introduction

Lidocaine is currently the most studied local anaesthetic in dentistry. Its pharmacodynamic characteristics are the baseline in comparative studies with other local anaesthetics.

Currently, all of these have an amide bond that gives them their characteristic safety parameters for latency, potency and toxicity.

Articaine falls within this category, except that it contains a thiophene ring within its chemical structure that gives it an increased solubility coefficient, resulting in faster diffusion over nerve structures (1).

The primary studies comparing articaine with other anaesthetics have primarily been performed in root canal surgery. References to oral surgery are even more scarce and primarily are concerned with tolerance (2-5).

The objectives of this study are to evaluate the anaesthetic properties and tolerance of articaine versus lidocaine at equal vasoconstrictor concentration. 

## Material and Methods

This study, approved by the Clinical Trials Ethics Committee (EudraCT No “2006-00303170”), was designed as a parallel, simple blind, single-site study with randomisation in four-element blocks or two treatments and was carried out in the Department of Medicine and Orofacial Surgery at the Complutense University of Madrid School of Dentistry. The study was open to the investi-gators and blind to the patients. 

A total of 96 male and female patients participated who underwent surgical treatment of the lower third molar. Patients were informed of the characteristics and objectives of the study and Informed Consent was obtained. Additionally, the current and previous medical and dental histories were obtained and compliance with the inclusion and exclusion criteria was established ( Table 1). 

6 blocks of 4 possible treatments were established; Test-A and reference-B ( Table 2). Treatment test-A used 1:100,000 articaine hydrochloride with epinephrine, injectable solution, marketed by Normon Laboratories S.A. under the brand name Ultracaine® (72 mg of articaine and 18 mg of epinephrine in 1.8 ml). Reference treatment-B used 1:100,000 lidocaine hydrochloride with epinephrine, injectable solution, marketed by Clarben Laboratories under the brand name Octocaine® (36 mg of lidocaine and 18 mg of epinephrine in 1.8 ml).

Using the conventional nerve root technique, 1.8 ml of anaesthesia was administered to block the inferior alveolar nerve and the lingual nerve. Once the first signs of labial numbness appeared, anaesthesia of the buccal nerve by administering 0.9 ml from a second carpule. Those cases that required administration of higher quantities of anaesthetic were excluded from the study. 

For surgical treatment of the lower third molars, an angular incision was made with mucoperiosteal detachment, eliminating the osseous surface that covers the third molar. Once the third molar was removed, the mucoperiosteal flap was

**Table 1:**
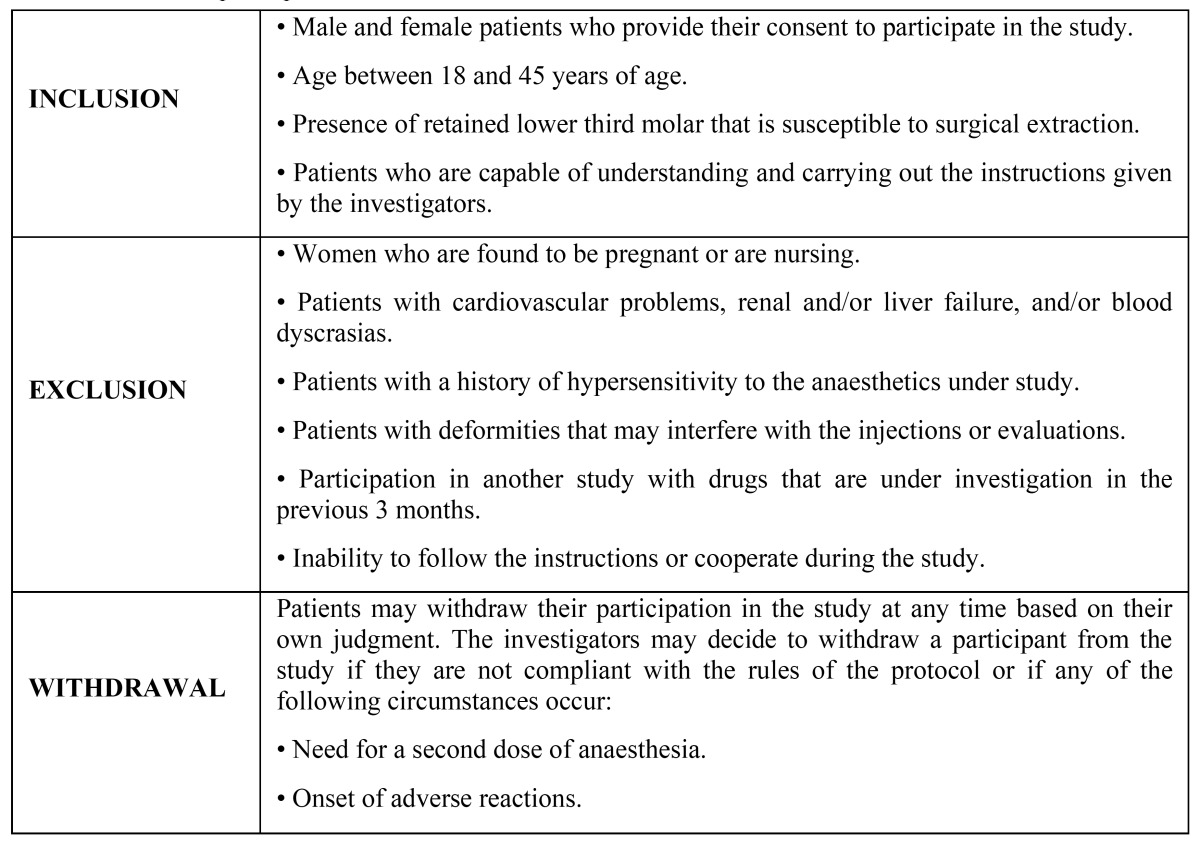
Selection of participants.

**Table 2:**
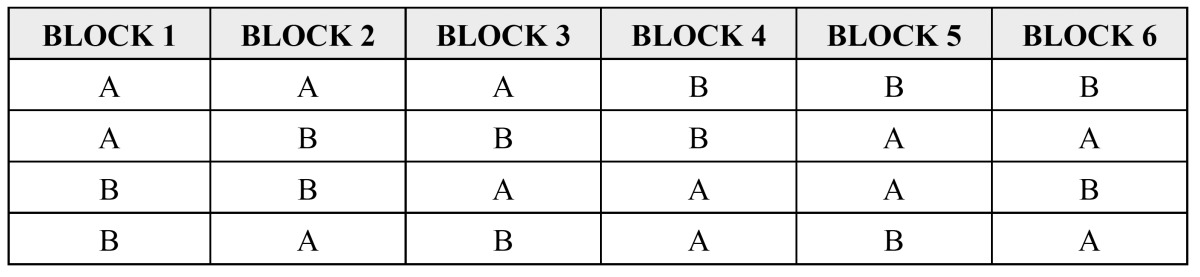
Randomisation blocks. Method for assigning volunteers to each treatment group.

**Table 3:**
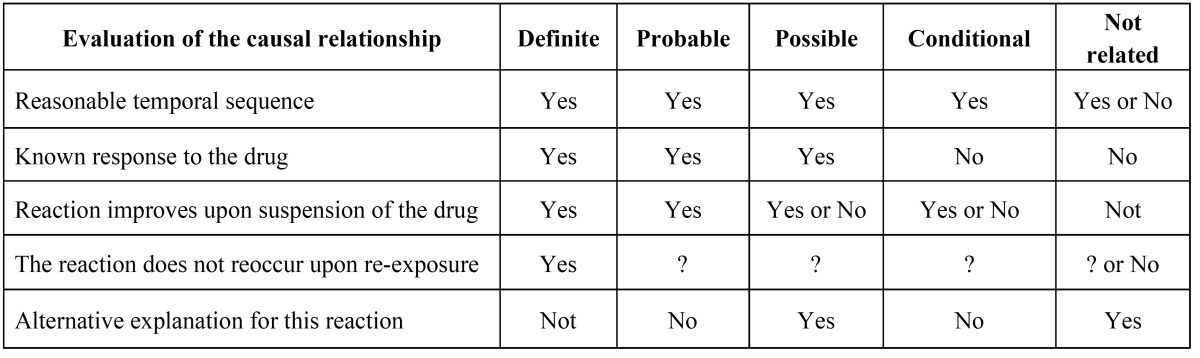
Adverse events based on the Karch and Lasagna algorithm (6).

repositioned with 000 silk and the patients were instructed on the normal postoperative recommendations.

Monitoring of the variables analysed intra-operatively consisted of:

Evaluation of latency time. Period in minutes from the time of administration of the local anaesthetic until the onset of the sensation of labial numbness.

Evaluation of the duration of anaesthesia. Patients recorded the time at which the sensation of anaesthesia (numbness) disappeared in the lower lip in their case report forms (CRF).

In order to analyse tolerance of both anaesthetics and the possibility of adverse events during the study, the patients were evaluated intra- and postoperatively for the onset of adverse events. These were noted in the CRF and were reported spontaneously by the patients. Each adverse event was described based on the moment of onset, duration, intensity, course and outcome in order to perform an evaluation of the causal relationship between the adverse effect and the medication. The intensity was evaluated based on a three-level subjective scale (mild, moderate, severe). The possible relationship of the adverse events to the study medication was classified as definite, probable, possible, conditional or unrelated according to the Karch and Lasagna algorithm (6) ( Table 3).

Based on the severity of the adverse reactions, when they occurred, they were classified as: severe – required continuous medical attention and/or loss of work; moderate - required administration of additional medication; and mild – no additional treatment required. 

The onset of any suspected adverse reaction should be reported immediately to the study monitor and the centre’s Clinical Trials and Research Committee (CTRC, San Carlos Clinic, Madrid).

Statistically, the overall descriptive results were expressed, depending on their characteristics, as frequency tables or as mean values ± standard deviation.

The anaesthesia latency time and the duration was evaluated using the ANOVA test with a 95% confidence interval with the anaesthesia used as the independent variable.

## Results

There were no significant differences in the duration of the intervention in those patients who received articaine or lidocaine (mean difference -10.69 seconds ± 1 minute 4 seconds, 95%CI: -2 minutes 19 seconds - 1 minute 57 seconds).

**Figure 1:**
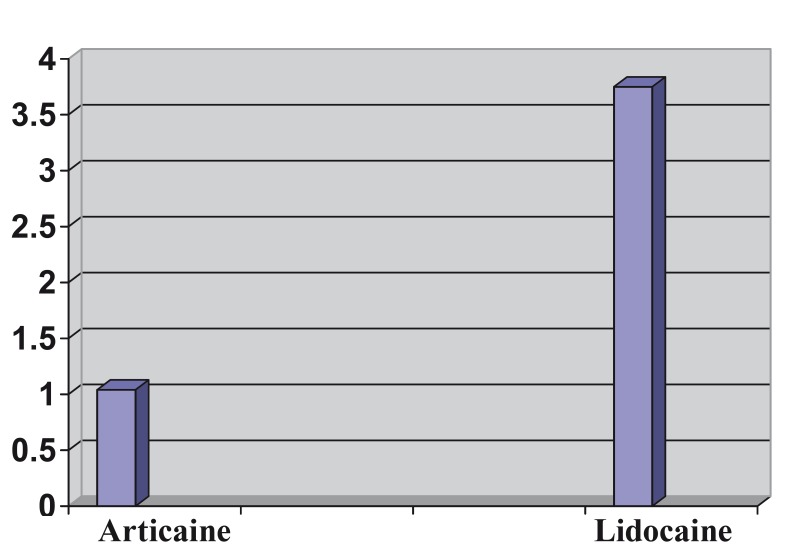
Latency period for articaine versus lidocaine, expressed in minutes.

Regarding the differences in latency time, though it was faster in the case of articaine, the differences were not statistically sig-nificant (1.04 ± 0.7 minutes for articaine versus 3.75 ± 14.71 minutes for lidocaine, with a mean difference of 2.70 ± 2.12 minutes, 95% CI 1.51 minutes - 6.92 minutes (Fig. 1). 

The duration of the anaesthetic effect, recorded by patients after the disappearance of the sensation of numbness in the lower lip, was also greater for articaine but the difference also was not statistically significant (4 hours 6

**Figure 2:**
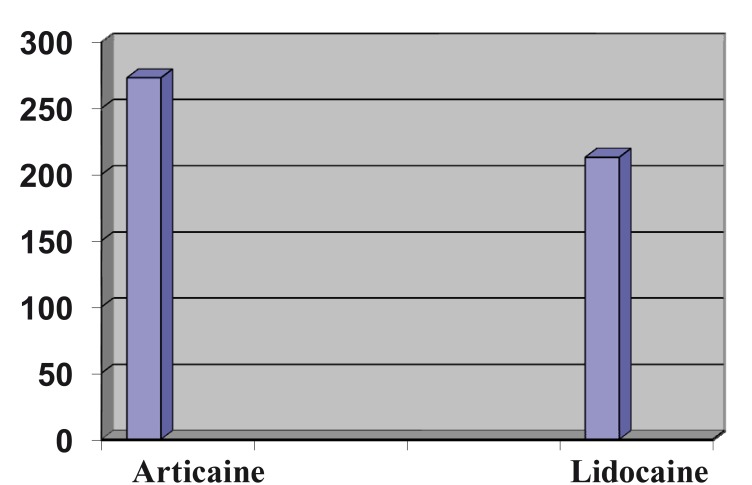
Time of anaesthesia duration for articaine versus lidocaine, expressed in minutes.

**Table 4:**
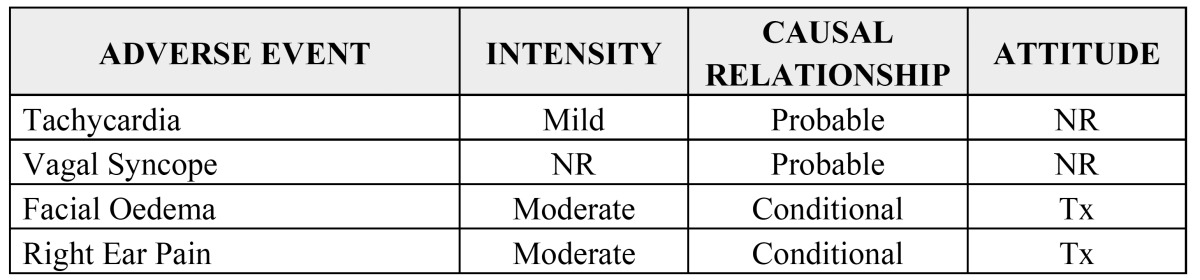
Adverse events. NR: Not recorded. Tx: treatment.

minutes ± 2 hours 28 minutes for articaine, compared with 3 hours 33 minutes ± 2 hours 35 minutos for lidocaine, with a mean difference of -33 minutes 5 seconds ± 31 minutes, 95% CI of -1 hour 35 minutes - 29 minutes) (Fig. 2).

Regarding the appearance of adverse events during the study, there were only instances in 4 patients. All were documented in accordance with the rules established in the study protocol, were self-limiting, and did not require the volunteers to be excluded from the study (Table  Table 4).

One male patient suffered an episode of tachycardia followed by hypotension and bradycardia with sweating (vagal syncope). The episode was mild in intensity, self-limiting following administration of the drug (articaine) and resolved spontaneously. Initially, a causal relationship to the drug was not assigned in the CRF. However, follow-up evaluation of the causal relationship to the study medication has been established as “probable”.

The second patient, a woman who belonged to the articaine group, presented with facial oedema, pain and swelling in the right auricular region during the 7 days following the intervention, probably related to a local infectious process. This required a change in the antibiotic and an extension of treatment with anti-inflammatory and analgesic medications. Initially, the causal relationship was considered conditional and the intensity was considered moderate. 

None of the adverse effects that occurred during the study were severe and none required withdrawal of the patient.

## Discussion

Lidocaine is probably the most widely-used anaesthesia in dentistry. Numerous clinical trials have been conducted in which it has been compared to other amide anaesthetics (7-10). The model applied to evaluate the variables of this study corresponded to surgery of the lower third molars. Multiple studies that use articaine have been analysed but these do not analyse its level of efficacy (11-13).

The first variables studied in this investigation, latency time and duration of anaesthetic effect, acquire a certain level of relevancy in patients who undergo surgery and who also have a certain level of anxiety as the speed of anaesthetic onset and duration of anaesthetic effect provides greater peace of mind.

For the first variable, latency period, no statistically significant differences were seen between both anaesthetics, though articaine was faster in achieving the first signs of labial numbness. 

These results concur with those found by other authors, establishing a mean value for this variable of around 1.2 – 2.5 minutes (14-16).

The duration of the anaesthetic effect also did not show any statistically significant differences, though greater duration times were seen for articaine. Authors such as Sierra et al. and Tofoli et al. 14-17) coincide with our results in establishing a mean interval of duration of approximately 245 minutes. However, the first of these, in their comparative study between articaine and lidocaine, statistically significant differences were found in favour of articaine.

Regarding the appearance of adverse events, there are few specific studies and they are occasionally subject to interpretation. Malamed et al. (18), in a multisite study in patients between 4 and 80 years of age performed in 2000 on 882 individuals who were anesthetised with articaine versus 443 with lidocaine, established that articaine, as with other amide anaesthetics, was safe and provided reliable anaesthesia during any dental treatment.

In 2008, Adewumi et al. (19), studying a series of 204 paediatric patients between 2 and 14 years of age, found the most common adverse effect to be injury to the lips due to biting. We confirm this effect, though it has not been the object of the study. In our opinion, we never recommend the use of articaine in child patients due to its prolonged anaesthetic effect on soft tissues, a situation that has been corroborated in our results.

We also have not found, in our experience nor in this study, temporary or permanent effect or injury to the inferior alveolar nerve as other authors have stated. We believe that these effects are difficult to attribute exclusively to the anaesthetic effect and may be attributable to the traumatic effect of the anaesthesia needle itself. The study performed by Pogrel et al. (20) in 2007 is interesting in that is established the same percentages for this reaction for lidocaine and for articaine.

The adverse events seen in this study were infrequent, of little consequence, and at no time required withdrawal of the patients.

The first of these, as has already been mentioned, developed a vagal response of rapid onset and rapid resolution. When interpreting this finding, one should also take into account the anxiety factor that many patients suffer when they are to undergo dental treatment. However, probably more importantly, is that it is related to the surgical intervention. Oral surgery is one of the surgical procedures that generates the greatest stress for patients. Additionally, the cardiovascular reactions that result from this situation of psychological stress vary between people and situations, which may produce cardiac or peripheral vascular reactions (21-24).

The second patient persisted with oedema and pain for a longer period than normal. It is also evident that the triad of pain, in-flammation and trismus is usually present following surgery of the lower third molars. There are numerous studies that state that the symptom of pain appears predominantly between 6 and 12 hours following surgery, though it may be mild during the first 48 hours. The inflammatory process is more prolonged, reaching 96 hours (25-27). 

The persistence of these symptoms beyond the expected time must be interpreted as a sign of active infection and therefore we should question the efficacy of the antibiotic treatment being administered. This is the circumstance that required us to change from amoxicillin to clindamycin in our case. This patient’s reaction was controlled at 10 days, returning the progression back to normal. This event was classified as conditional because this occurred during a clinical trial and, as a result, one is required to record any event that occurs in the CRF.

In summary, we can state that both articaine and lidocaine have demonstrated and adequate and similar safety and tolerability profile. For this reason, their use in oral surgery should remain at the discretion of the professional who will evaluate their use based on the necessary surgical time.
